# What are the strategies for implementing primary care models in maternity? A systematic review on midwifery units

**DOI:** 10.1186/s12884-022-04410-x

**Published:** 2022-02-14

**Authors:** Laura Batinelli, Ellen Thaels, Nathalie Leister, Christine McCourt, Manila Bonciani, Lucia Rocca-Ihenacho

**Affiliations:** 1 Centre for Maternal and Child Health Research, School of Health Sciences, City, University of London, 1 Myddelton Street, London, EC1R 1UW UK; 2 Faculty of Health & Wellbeing, School of Community Health and Midwifery, University of Central Lancashire, UCLAN, Brook Building, Victoria Street, Preston, PR17QT UK; 3 Laboratorio Management e Sanità, Institute of Management, Scuola Superiore Sant’Anna, Piazza Martiri della Libertà, 33, CAP 56127 Pisa, Italy

**Keywords:** Midwifery units, Midwifery led care, Birth centres, Midwifery centres, Primary care models, Implementation, Innovation, Adoption, Metasynthesis, Qualitative research

## Abstract

**Background:**

Midwifery Units (MUs) are associated with optimal perinatal outcomes, improved service users’ and professionals’ satisfaction as well as being the most cost-effective option. However, they still do not represent the mainstream option of maternity care in many countries. Understanding effective strategies to integrate this model of care into maternity services could support and inform the MU implementation process that many countries and regions still need to approach.

**Methods:**

A systematic search and screening of qualitative and quantitative research about implementation of new MUs was conducted (Prospero protocol reference: CRD42019141443) using PRISMA guidelines. Included articles were appraised using the CASP checklist. A meta-synthesis approach to analysis was used. No exclusion criteria for time or context were applied to ensure inclusion of different implementation attempts even under different historical and social circumstances. A sensitivity analysis was conducted to reflect the major contribution of higher quality studies.

**Results:**

From 1037 initial citations, twelve studies were identified for inclusion in this review after a screening process. The synthesis highlighted two broad categories: implementation readiness and strategies used. The first included aspects related to cultural, organisational and professional levels of the local context whilst the latter synthesised the main actions and key points identified in the included studies when implementing MUs. A logic model was created to synthesise and visually present the findings.

**Conclusions:**

The studies selected were from a range of settings and time periods and used varying strategies. Nonetheless, consistencies were found across different implementation processes. These findings can be used in the systematic scaling up of MUs and can help in addressing barriers at system, service and individual levels. All three levels need to be addressed when implementing this model of care.

## Background

A growing body of evidence has identified the impact and cost-effectiveness of midwifery models of care in improving maternal and newborn health [1, 2]. The Lancet series on Midwifery highlighted the central role of midwifery care models in preventing the “too much too soon and too little too late” phenomenon that is affecting maternal and newborn health worldwide, both in low- and middle-income countries (LMIC) and in high income countries (HIC) [3, 4].

International studies have demonstrated that for healthy women with uncomplicated pregnancies, midwifery units (MUs) are associated with better maternal and similar perinatal outcomes compared to obstetric units (OUs) while being cost-effective and associated with high satisfaction amongst service users and midwives [5–7]. MUs were mapped in over 56 LMIC and HIC countries on the Goodbirth.net platform [8]. The Midwifery Unit Standards for Europe (2018) and the commentary by Stevens and Alonso (2020) helped in reaching consensus of the definition and the standards for MUs in different international contexts [9, 10]. The MU standards for Europe defined a midwifery unit as “*a location offering maternity care to healthy women with straightforward pregnancies in which midwives take primary professional responsibility for care. Midwifery units may be located away from (Freestanding) or adjacent to (Alongside) an obstetric service*” [9]. Stevens and Alonso (2020) expanded this definition for LMIC to also include sexual and reproductive health as part of the main midwifery centre activities [10].

Walsh et al. (2020) recently published a study about which factors affect the implementation and improvement of MUs in England and highlighted an underutilisation of this model of care even in a country with a long history of policy and guidelines supporting MUs [11]. However, there is still little international literature on how to implement MUs in contexts in which the OUs represent the main form of care provision.

The main aim of this review is to identify and synthesise existing knowledge on how to support the implementation of new MUs internationally, to fill the evidence to practice gap and to learn from existing evidence on how to support this change of the maternity care provision in the real world. The research question chosen for this review informed by a scoping search was: *“What are the strategies used for implementing new midwifery units internationally?”.* This review is the first of its kind.

## Methods

The “*Guidance on choosing qualitative evidence synthesis methods for use in health technology assessments of complex interventions*” informed our methodology decision [12]. The following points for each type of methodology were considered to decide which type of review to conduct: type of review question, epistemology, timeframe, resources and team expertise. The thematic synthesis method by Thomas & Harden (2008) was selected [13]. This method was developed to address review questions focused on need, acceptability and appropriateness of intervention which suits well the aims and nature of the review question of this review [13, 14].

This review was not focused on clinical outcomes of MUs. Instead it aimed to understand implementation related outcomes like acceptability, adoption, appropriateness, costs, feasibility, fidelity, penetration and sustainability, as defined by the taxonomy of Proctor et al. (2011) [15].

This review was registered on the International Prospective Register of Systematic Reviews (PROSPERO) on the 18th of October of 2019 with registration number: CRD42019141443.

To conduct the search and screening, the PRISMA guidelines (Preferred Reporting Items for Systematic reviews and Meta-Analyses) were used [16] and the following inclusion and exclusion criteria were agreed (see Table 1).

**Table 1:** Inclusion and exclusion criteria

	Inclusion	Exclusion
Participants	All stakeholders involved in implementing midwifery units: maternity teams, health institutions, professionals, service users	Models of care not specific to midwifery, birth settings managed or led by obstetricians or other healthcare professionals other than midwives, home births
Phenomenon of interest	The process of implementation of a new MU which could be successful or not. For successful implementation we mean the establishment of a new MU after a process of change in the maternity care setting.	Focus on improvements of existing MUs Focus just on clinical outcomes or technical quality of care. Focus on specific issue (e.g. smoking cessation, vaginal birth after caesarean - VBAC).
Outcomes	Implementation outcomes like acceptability, adoption, appropriateness, costs, feasibility, fidelity, penetration and sustainability.	No focus or substantial data on questions relating to implementation, sustaining and uptake or scaling up.
Study design	All designs including action research, grounded theory, ethnography, mixed methods studies that include qualitative data collection and analysis.	No restrictions on the types of study design were applied.
Study focus	Studies will need to cover aspects related to implementation outcomes in the data collection and analysis with particular attention to any relevant aspect or strategy related to the establishment of a new MU.	Clinical or technical quality of care. Focus on specific health issue (e.g. smoking cessation, VBAC)**.**
Setting	Both alongside (AMU) and freestanding (FMU) midwifery units. Birthing rooms physically/organisationally separated from the main OU. Maternity systems willing to/in the process of implementing a new MU. Private and public services All countries	None
Time period	No time restriction	
Language	English, Italian, Dutch, Portuguese, Spanish, French	Other languages that the team would not be able to translate adequately.
Publication type	Peer reviewed articles Dissertation and theses Research reports	Any piece of research which cannot be peer reviewed by the research team (books, opinion pieces, commentaries, diaries etc.)

### Systematic search and screening process

The systematic search was conducted between December 2020 and April 2021. Databases searched for this review were: Ebsco Databases (Medline, CINAHL, SocINDEX), Ovid databases (Embase, Global Health, Maternity and Infant Care MIDIRS, Ovid Nursing, Ovid Emcare), Scopus and NICE database. Grey literature was searched via OpenGrey, Google Scholar and ProQuest Dissertation and Theses.

The final strategy applied to each database is reported in Table 2.

**Table 2:** Search strategy modified the terms

Search terms:	Order	Search strings
Implementation	1	Mesh terms for implementation
	2	Keyword search: implementation OR implement* OR “knowledge translation” OR innovation OR utili#ation OR “scale up” OR feasab* OR sustainab* OR “service improvement” OR barrier* OR facilitator* OR enabler* OR adopt* OR diffusion OR establish* OR open* OR transition OR provision OR embed* OR integrat* OR planning OR preparation OR “implement* strategy*” OR promot*
	3	1 OR 2
Midwifery units	4	Mesh terms for midwifery units
	5	Keyword search: “midwifery unit” OR “midwi* led birth* cent*” OR “birth* unit” OR “birth* cent*” OR “birth setting” OR “low risk birth* cent*” or “midwi* unit “OR “midwi* led unit” OR “low-risk birth* room*” or “midwife-led room* “OR “midwi* cent* “OR “low-risk birth* cent*” OR “homely birthplace” OR “homely birth place” OR “homely birth* room*” OR “normal birth* unit”
	6	4 OR 5
Full search	7	3 AND 6

The research team added some key relevant articles to the search on the databases and conducted a citation track referencing. After de-duplication, the papers identified were saved and divided in three sub-folders so that LB, ET and NL could run a screening by title and abstract for relevance and against pre-determined inclusion and exclusion criteria. The team met regularly to discuss papers and reach agreement in the screening. Any cases where agreement could not be reached were discussed with CMcC (author and senior researcher). This process was then replicated by reading full texts of articles selected as potentially relevant.

## Search results

**Fig. 1:**
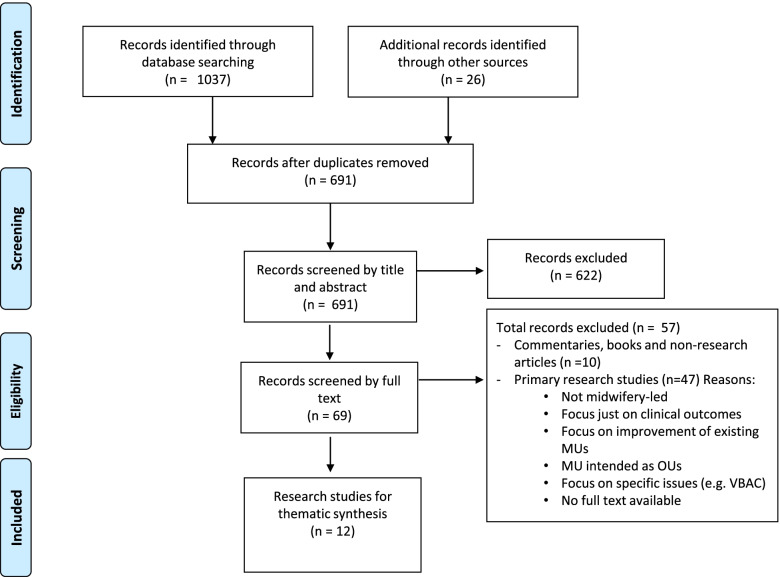
Screening process using PRISMA flowchart

After a systematic search, a total of 1037 articles were identified and 26 papers were added after citation track referencing, ending up with the identification of 1063 articles. After de-duplication, 691 papers were screened as shown in Fig. 1.

Of the sixty-nine studies selected for full text screening, only twelve studies were primary research and eligible for the aims for this review. One good quality study (10/10) about AMUs in England was included twice [17, 18] comprising a peer-reviewed journal article and a more in-depth report rich in useful data. In Table 3, they are listed as 9A and 9B to clarify this. The Chinese and the Brazilian case studies had two papers each related to different aspects of the implementation process. Therefore, we listed them as 1A/1B for the Chinese and 4A/4B for the Brazilian (see Table 3). The quality of the studies identified was overall good with scores above 6/10 and five studies scored 10.

**Table 3:** Characteristics of included studies

N	Author, Year	Country	Study aims	Design	Participants	Setting and data collected	Findings	Quality
1a	Cheung NF et al. 2009 [19]	China	To describe the preparations for setting up a midwife-led normal birth unit which was based on literature and practice review	Action research with a five steps cycle plus a literature review	8 midwifery team leaders 5 researchers	A highly medicalised maternity department in a Chinese hospital with annual birth rate of over a 3000. The MU was allocated two birthing rooms. The researchers analysed data from meetings, field notes and midwifery training course.	The findings are divided into seven sections: definition, negotiations, accommodation, specific practices, the philosophy of the homely birthplace, policy development, and developing local solutions for local aspirations.	8
1b	Mander R et al. 2009	China	To explore issues arising during preliminary stages of the action research project to consider the feasibility and the effects of a MU on midwives and women.	Action research using a qualitative descriptive approach	Non-defined number of stakeholders including midwifery staff, managers, university staff and researchers.	(*same setting as above*) Data were collected at meetings, by non-participant observation and by face-to-face semi-structured interviews.	MU care may be feasible after the analysis of the early stages of implementation.	8
2	Mackey MC et al.1991 [20]	US	To report on how the idea of birthing room was initiated by nurses and the 8 strategies that led to the implementation of it.	Structured interviews	4 registered nurses with Master’s degree	Four private hospitals located in the Chicago area. One-hour in-depth interviews.	Eight strategies to be used jointly to open new birthing rooms by nurses’ midwives	7
3	Moudi Z et al. 2013	Iran	To assess 10 years of experience of the first Safe Delivery Posts (SDPs) established in Zahedan, Iran and to examine the reasons why women chose to give birth there.	A mixed-methods research	19 service users in the postnatal period	The two SDPs in Zahedan, the most populous city in the province. Women were selected from two Safe Delivery Posts in Zahedan city in southeast Iran.	Implementing a model of midwifery care that offers the benefits of modern medical care and meets the needs of the local population is feasible and sustainable. This model of care reduces the cost of giving birth and ensures equitable access to care among vulnerable groups in Zahedan.	9
4a	Pereira AL and Moura MA 2009 [21]	Brazil	To identify the determinants of the process of implementing the Birth Center and analyse the influence that hegemonic and counter-hegemonic groups have on that process	Dialectic qualitative research	4 commissioners 11 technical administrative professionals	Casa de Parto in Rio de Janeiro. Individual semi-structured interviews.	During the establishment process, conservative and transformative forces of the hegemonic childbirth care model clashed in the governmental and civil spheres. Legal and political dispute in the establishment process of the Casa de Parto highlighted the importance of organized social movements, especially the women’s movement.	7
4b	Progianti JM et al. 2013 [22]	Brazil	To discuss how the Brazilian nurse midwives trained in the Japanese birthing centres helped to implement the FMU in Brazil.	Socio-historical study with qualitative approach	1 Director of nursing 1 Nurse midwife 1 Physician 1 Former nursing director	Casa de Parto in Rio De Janeiro. Written and oral documents. Semi-structured interviews and report of the exchange experience. Data triangulation with policy and background documents.	The exchange programme enabled the Brazilian midwives to implement the first MU in Rio de Janeiro and added a larger volume of capital to their professional habitus.	9
5	Reszel J et al. 2018 [23]	Canada	To obtain the perspectives of health care providers and managerial staff about the integration of the new FMUs one year after implementation	Qualitative descriptive approach	24 amongst professionals (18) and managerial staff (6)	Ontario where homebirth and birth in OU were the only two birth settings for women prior the implementation of the two FMUs. Data was collected via 4 focus groups and 1 interview.	The collaborative approach for the planning and implementation of the MUs was a key factor in the successful integration and the positive experience of service users.	10
6	Walton et al. 2005 [24]	England	To explore organisational factors, midwives role, barriers and facilitators of the change process and training needs for midwives	Action research	Non-defined number of stakeholders including midwives, managers and medical staff.	Inner London teaching hospital that take care of over 4400 women a year. Data from meetings, educational workshops, feedback forms and audit of the 2 birthing rooms	The lack of support from medical staff, the conflicting priorities and the dominance of the medical model of care made the project not feasible and the team abandoned the idea of the MU after this pilot.	6
7	Walsh et al. 2018 [25]	England	To describe the configuration of midwifery units, both alongside & freestanding, and obstetric units in England	National survey	Heads of Midwifery in English Maternity Services	National Health Service (NHS) in England. Descriptive statistics of AMUs, FMUs and OUs and their annual births/year in English Maternity Services	Number of MUs and births in MUs in England increased after the publication of NICE guidelines (mostly AMUs). Significant difference in terms of utilisation of the MU and this suggest that some are underutilised.	10
8	Walsh et al. 2020 [14]	England	To identify factors influencing the provision, utilisation and sustainability of MUs in England	Qualitative study	57 Obstetric, midwifery and neonatal clinical leaders, managers, service user representatives and commissioners 60 midwives 52 service users	Setting England. Data collected: first, MU access and utilisation across England was mapped; second, local media coverage of the closure of free-standing midwifery units (FMUs) were analysed; third, case studies were undertaken in six sites to explore the barriers and facilitators that have an impact on the development of MUs; and fourth, by convening a stakeholder workshop.	Most managers and clinicians did not regard their MU provision as being as important as their OU. The analysis illuminates how implementation of complex interventions in health services is influenced by a range of factors including the medicalisation of childbirth, perceived financial constraints, lack of leadership and institutional norms protecting the status quo.	10
9a	McCourt et al. 2018 [26]	England	To investigate how AMUs are organised, staffed and managed, the experiences of women, and maternity staff including those who work in AMUs and in adjacent obstetric units. Some MUs were already established, other just recently being implemented.	Organisational ethnography approach	35 managers and key stakeholders 54 professionals 47 service users	Case studies of 4 AMUs in England, selected for maximum variation based on geographical context, length of establishment, size of unit, leadership and physical design. Observations, semi-structured interviews and documentary review were conducted.	Development of AMUs was often opportunistic. Key potential challenges included: boundary work and management; professional issues; developing appropriate staffing models and relationships; midwives’ skills and confidence; and information and access for women.	10
9b	McCourt et al. 2014 [18]	England	(*same as above*)	(*same as above*)	(*same as above*)	(*same as above*)	Same as 9A but explored more in detail.	10

### Quality appraisal

Two independent reviewers (LB and ET) carried out critical appraisal using the CASP Critical Appraisal Skills Programme Qualitative Research Checklist (CASP) [26] and any differences at any stage were discussed with a more senior team member (CMcC). A simple scoring system was added to this process to assist in summarising quality level. Each study was rated zero or one for each item of the CASP question if it was fulfilling the requirement or not (1 = yes, 0 = no). Every time that the score was “0” the reason for that score was reported. The sum of all CASP questions constitutes the quality score of the study (1 to 10). During the writing of the synthesis, the team used a sensitivity analysis and more importance was given to the higher quality articles.

### Data analysis and synthesis

The articles selected for the analysis were imported into NVivo 12 software for data analysis. Data in the abstract, findings and discussion sections were analysed thematically using a three-stage process approach: coded line-by-line, organised into categories to capture descriptive themes and analytical themes were then developed to answer the review questions [13].

### Descriptive findings

The studies selected were conducted in England, Brazil, China, Canada, Iran and United States (US). Seven studies were published between 2010 and 2020 when more substantial evidence on outcomes of MUs was available, five studies took place between 1991 and 2010. Healthcare systems in different contexts and time varied quite significantly amongst the studies. A public system with universal coverage was present in countries like England and Canada whilst a mixed system with public governmental system, private sector, and NGOs was present in Brazil and China, Iran, and US.

Some studies were not purely focused on the implementation process of a new MU [11, 17, 18, 25, 27], but had wider aims such as mapping MUs nationally or investigating how AMUs were organised. However, the team could identify interesting and relevant aspects related to implementation of new MUs in these studies and therefore included them in the analysis.

This study aimed to analyse quantitative and qualitative data however only three studies included a quantitative component in their research design [24, 25, 27]. Two of them [24, 27] used quantitative data to describe the use of the MU after implementation (i.e. number of births per year) and not the implementation process therefore they were not relevant to the aim of this review. The last one, by Walsh et al. (2018), described the change of the maternity service configuration after the Birthplace study in England and the impact that this had in the adoption of MUs there. Since 2011 and the publication of the NICE guideline 2014 which were recommending for the first time the option of giving birth in a MU to all women with an uncomplicated pregnancy, the number of AMUs increased from 53 to 97 and the FMUs from 58 to 61. The number of Trusts (organisational units within the English National Health Service) without a MU significantly decreased from 75 to 32.

Midwifery was less regulated and less autonomous in countries like China, US and partially in Brazil with higher level of autonomy reported in England and Canada. No information on the status of midwifery was available in the Iranian study [27].

There was variability with the MU model of care within different countries. The common characteristics across all sites were: an intrapartum unit (within the OU, alongside or freestanding but always physically separated from the main OU rooms) staffed by midwives (hospital or community midwives) who worked autonomously providing a midwife-led primary level of care and referring service users to the secondary level of care (in situ or via transfer) when needed.

In most of the studies, participants were mainly professionals, managers and commissioners. Service users were included just in four studies and three of them were based in England.

### Synthesis findings

The discussion of the synthesis is presented under two broad categories: readiness (elements found to be important in the local context at the beginning of the implementation process) and strategies (main actions and key points identified in the case studies selected). The first category is divided into cultural, organisational and professional levels whilst the latter includes four key themes, each of which covers common strategies, barriers and facilitators to the change.

**Fig. 2:**
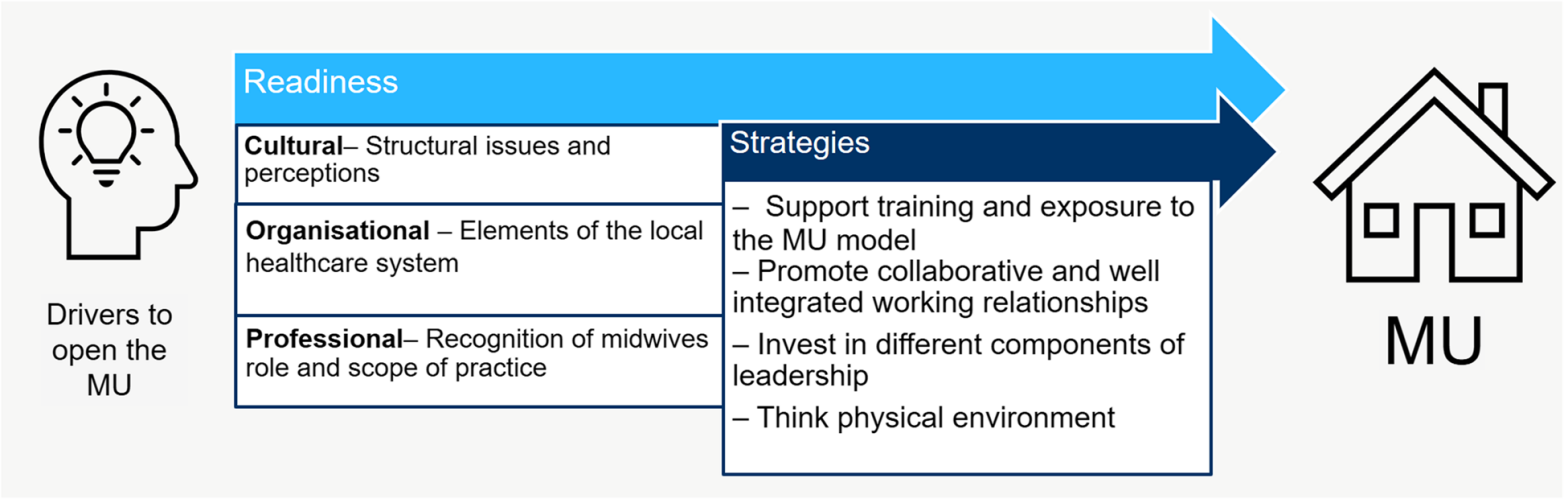
Logic model

In Fig. 2, a synthesis of the emerging themes are presented in a logic model composed of two main categories: readiness and strategies. This model was created to give a temporal and visual idea of the different role that these themes have during an implementation process. From the initial idea of opening a new MU to the actual adoption of the model a multi-layered change needs to take place.

## Readiness

### Cultural level - structural issues and perceptions

#### Structural issues

Codes related to culture and perceptions were ubiquitous across the different articles showing that all participants discussed on some level aspects related to society, the local culture and how this affected the implementation process. Studies took place across seven countries with differing healthcare systems and periods of time when the implementation was attempted, however some consistencies were found.

On a macro-societal level, structural issues highlighted as barriers were related to gendered power dynamics, hierarchy in the health system and the hegemonic production logic in healthcare [11, 17, 18, 21]. For example, in the study by McCourt et al. 2014, professionals described an unbalanced gendered dynamic as a barrier to implementation and to the existence of AMUs [17]. Amongst the different countries, women have different levels of autonomy, respect and rights when it comes to childbirth. The case studies from Brazil, China and Iran discussed the issue of women’s rights in childbirth and obstetric violence acknowledging its presence in the respective countries [19, 21, 27, 28]. Opening new MUs became an opportunity to tackle this issue and the following quotes from the Iranian study shows how the MU was perceived by service users as valid alternative to avoid such mistreatments:

“*I have insurance. If I had gone to hospital, it would have been free of charge for me, but I didn't. They annoy us in hospital; they examine too much. It's more comfortable here; it's better.”* Service user, [27], page 1078

The information provided to women about choice of place of birth played a key role in the decision-making process that was often found to be rigid. An example of this was asking service users to decide where to give birth at the very first booking appointment [17, 18] with not many occasions to reconsider their choice. This rigidity was also mentioned in the Chinese studies [19, 28].

The medicalised and industrialised model of care was cited in the English and in the Brazilian studies as a structural problem that can become the key obstacle to implementation [11, 18, 21, 24]. These studies identified that in a system that functions with a hierarchical structure and in terms of efficiency and productivity, the division between the Industrial/Medical model of care of the OU and the Bio-Psycho-Social model of care of the MU [9, 29] could lead to polarisation, with an imbalanced power dynamic.

“*A normatively medical outlook persisted, that located midwifery units as marginal rather than as a core maternity service.”* Authors, [18] page 18

In this scenario the OU represented the priority of the service and the MU an alternative which could be closed if need be.

#### Norms and perception of safety

A significant part of participants’ quotes was about perceptions of safety. The English studies identified that the MU being co-located in the same building was perceived to be safer than FMUs [11, 17, 18, 25]. This was often mentioned by participants (both professionals and service users) even though it is not supported by existing literature that shows that FMUs are instead associated with better clinical outcomes than AMUs [6, 30].

*“I think majority of women and all my friends will opt for an alongside MU, because most women do want the option of midwifery led but if anything goes wrong they just want to go down that corridor, through that door.” Midwifery Manager,* [11]*, page 5*

Some professionals also mentioned the idea of feeling safer by having all women in the same place and therefore having greater monitoring (and control) than having them in different locations. This preconception was illustrated in this quote by an English consultant obstetrician:

*“(…) if I were to design a unit I wouldn’t split my shop in two different places on the high street. It just doesn’t make sense to me. If you have everybody all in one place you don’t have those problems. You’ve got greater monitoring of everything that’s going on; you’ve got greater use of your resources, [it’s] more efficient” Consultant obstetrician,* [17]*, page 22*

On the other hand, when professionals were educated and had knowledge on the evidence and the impact that a MU might have, there was better integration and working relationships. This seemed to show the importance of information and education of best available and up to date evidence to make stakeholders aware of the impact of MUs on social and clinical outcomes and cost-effectiveness.

In the Iranian case study, choice was often about compromising on what was affordable [27]. It was noted that women often reported perceiving the OU to be safer than the MU because of the availability of medicines and devices. However, they would opt for the MU to access a good level of care by experienced professionals at an affordable price.

*“I thought, childbirth is just childbirth, no matter which place I go to. Why should I go to hospital, where the costs are very high? I didn't have health insurance, and I had to pay all that money in cash (out of pocket). Therefore, I decided to go to the nearest SDP (MU)” Service user,* [27]*, page 1078*

The MU constituted the best compromise for that population to gain physical and psychological safety. However, the MU represented also the birthplace option that would allow them to avoid unnecessary medicalisation of childbirth:

*“I love my daughter-in-law very much. Her childbirth was a hard time for me. In hospital, they told me she needed a caesarean, so I took her to the Post (MU). I didn't tell the ladies here (midwives) what I had been told in hospital. And thank God she had a natural delivery.” Service user,* [27]*, page 1079*

### Professional level - recognition of midwives’ role and scope of practice

Most studies discussed the importance of a midwifery identity and the role that this profession had in those contexts. Midwifery and midwifery-led care was established with different level of autonomy. England and Canada had midwives that could practice autonomously in these units [11, 17, 18, 23–25]; Brazilian midwives went to Japan to gain more exposure of the midwifery model of care as they were not used to work with that autonomy [22], whilst China, US and Iran [19, 20, 27, 28] reported not having a well-established and autonomous midwifery workforce in the healthcare system at that time.

Contexts in which midwifery was not established as an autonomous profession seem to struggle more, especially in the first phase of the implementation when the idea needed to be accepted by other stakeholders [19, 20, 22]. In the Chinese case study, the opportunity of implementing a MU was reported to be the means to achieve a proper and recognised professional status [19].

The need of having obstetricians to promote a midwifery led model seemed important in all contexts but particularly so where midwifery was more marginalised in the decision making of the service configuration. However, it could have a ‘boomerang’ effect in which once the MU is implemented, the obstetric component could claim the leadership. In the American study, for example, marginalisation of the midwifery profession became apparent when nurse midwives who promoted and initiated the project of MUs had to fight with the obstetric component for the recognition and the credit of their actions:

*“Although nurses were the initiators of the birthing room (MU) concept and nurses did most of the work towards implementing the concept, there is evidence that physicians are pre-empting the credit. One nurse said, -It’s interesting that now the doctors think it’s their idea-. Another nurse was concerned that nurses never received credit for changes they had made in her hospital and tried to avoid a repeat of that situation.” Authors and nurse midwife quote,* [20]*, page 266*

The recognition of midwives’ role and scope of practice was needed not just within the organisation and amongst professionals but on a more societal level too. This was not limited to countries where midwives are less autonomous but also to countries like Canada, where professional establishment was relatively autonomous but still recent and small-scale. In this case, the MU became a facilitator for this process of recognition of the midwifery scope of practice and therefore promotion of its role in society:

*“Many participants perceived that the birth centers (MUs) have increased the respect and legitimacy of midwifery, both to the public and to other health care professionals, allowing these groups to learn more about midwifery and ultimately increase visibility and credibility of their education and practice. One paramedic stated, ‘It elevated the [midwifery] profession for sure . . . I think just having the facility speaks volumes to the interest, the buy-in, the respect, and the credibility of midwifery*’.” Authors and paramedic’s quote, [23], page 5462

### Organisational level - elements of the local healthcare system

#### Cost and financing systems

Study authors reported that the concept of cost effectiveness associated with MUs was not always clear to commissioners, managers and professionals [11, 17, 18]. The concept of MU being “cost-saving” was often mentioned together with the status of financial constraint and the urgent need for healthcare organisations to save money [11, 17–22, 27, 28]:

“*Financial constraints within Trusts were often seen as limiting the development of MUs. While economic evaluations suggest the overall economic outcomes of increasing births in MUs is positive, the start- up costs were seen as a barrier, and the longer term savings from lower morbidity in the target population that accrue across the health system were not recognised. In a climate of scarcity, new ways of structuring care must demonstrably save money, or at least, be perceived to, in the short term.*” Authors, [11], page 7

Studies identified two threads of opinions: one perceived MUs as expensive and unaffordable luxuries, or small and so inefficient [11, 17] and therefore an antithesis to the need of save money of the organisation; the other perceived the cost-saving attribute negatively as if this would necessarily mean a lower quality of care. In Brazil for example, this argument was used by the organisations which were against the promotion of MUs and in favour of a more medicalised approach; they referred to the MU model as “*poor care for the poor*” [21].

Managers, commissioners and professionals’ perceptions and willingness to implement the MU was also dependent on the type of healthcare system and commissioning environment of the local context. Where there was a ‘payment by results’ tariff in which the organisations were paid for the interventions provided, normal births were often seen as a “*loss making activity*” by commissioners and obstetricians [17–19]. In the US, where hospitals were paid by number of births, the strategy used by nurse midwives to convince physicians and commissioners that the MU would attract more women to their service was considered one of the most effective approaches [20]. In China, midwives were asked to take more responsibility working in a MU without an economic incentive, they were tempted to prefer working in the OU where for the same salary they had less responsibility [19]. In Iran, where service users had to pay depending on the place of birth they chose (MU or OU attended by professionals or homebirth attended by SBA), the MU offered services which were more affordable to them while ensuring good quality of care.

A financial system that was perceived working better in promoting midwife led provision and normal births was the one based more on assessment of risk level and service users’ needs at booking [17, 18]:

*“Although the commissioning environment and payment tariffs had been described as making normal birth a ‘loss-making’ (manager XXX) activity, managers and commissioners hoped that the development of a tariff centred more on assessment of women’s care needs would help to remove such perverse incentives.” Authors* [17]*, page 42*

#### National guidelines

In all the case studies contexts, giving birth in an institutionalised unit even if outside the main traditional OU was legal and this represented an important first step towards readiness for the change. A clear example of positive impact, as reported in one English study, were the NICE Intrapartum guidelines published in 2014 that were promoting MUs and the possibility for each woman to choose between 4 places of births based on the findings of the Birthplace Study [5, 11, 25, 31].

Similarly, in Canada and Brazil, the new national guideline promoting the MU model of care was reported as a key trigger for an implementation process towards MUs [21–23].

Guidelines also played an important role in professionals’ perception of safety and for the collaborative work of the multidisciplinary team [17, 18].

“*In XXX, for example, managers emphasised the need for obstetric support for normal birth and midwife-led care and saw guidelines as helping to sustain obstetricians’ confidence in the alongside unit. It was apparent that obstetricians were more comfortable with midwife-led care away from the obstetric unit if they felt that there was a comprehensive set of guidelines supporting that care that had been agreed across the service. This gave them more confidence that women would be appropriately referred to them for review if medical attention were necessary.” Authors,* [18]*, page 18*

Having a national guideline is a first step and a key facilitator for the implementation of these realities to allow local stakeholders starting a conversation around the adoption of the different model.

#### Local policies

The opportunity for a MU came often from the idea of revising or creating a new local protocol for physiological labour and birth. This promoted integration, as this example from an English study highlights:

*“Managers and midwives saw the local guidelines for admissions to and transfers from the midwifery unit as protecting a space for physiological birth, as well as a guide and framework for safe practice.” Authors, *[18]*, page 18*

On the other hand, attempting an implementation without such local guidelines could jeopardise the whole process leaving space to interpretation, no clear distinction in pathways of care and contamination of practices (as will be further discussed in point 4.2 of this review).

*“Midwifery units and midwives, as well as the women themselves, were perceived to be vulnerable without such guidelines, which also helped to create and protect a space for supporting physiological birth.” Authors,* [17]*, page 25*

When preparing a local protocol for the management and practice in the MU, key topics that needed facing and addressing were the access criteria of the MU and transfer criteria from the MU to the OU.

*“Prior to the opening of the birth center, we managed collaboratively with our key stakeholders, so we managed with the nurse manager but also some of the physicians, the obstetricians, about developing our current [transport] protocol . . . But it [was] something that we, from scratch, met together collectively, collaboratively to get everyone’s approval for the current protocol that we have.” Midwife,* [23]*, page 545*

The multidisciplinary exchange in the production of these criteria became an opportunity for collaborative practice and a facilitator to the MU's implementation.

## Strategies

### Support, training and exposure to the MU model

All studies identified that an appropriate set of knowledge, skills and training was required for midwives to work autonomously, even though midwifery regulations and background of midwives had significant differences from one context to another. Even studies located in countries where midwives worked more autonomously (England and Canada) reported a lack of confidence in physiological birth among midwives often due to a more predominant obstetric-led practice in the last decades:

*“Because everyone has worked in such a high-risk environment, you become deskilled to an extent, and feel a bit apprehensive about normal birth… you know, trusting that women can have babies low risk.” Focus Group Midwife,* [11]*, page 6*

A good level of knowledge, up to date training and appropriate skills of the midwifery workforce were identified as an important facilitator to develop professionals’ confidence in the MU model and for being able to promote it and spread it.

### Training

A strategy identified in all studies was supporting staff with training initiatives as an enabler of the change. In some cases, midwives identified their own educational needs prior the implementation of the MU model of care and this helped engaging them in the project and create sense of ownership [19, 22–24]. The autonomy and skills gained via the training helped increasing not just the clinical confidence but also the confidence in the midwifery scope of practice, the vision of the MU and its implementation [22, 23].

Ad hoc and pre-implementation training for midwives was promoted, but also the concept of regular training, the so-called continuous practice development (CPD), was addressed in several studies [11, 17, 18, 23]. Studies highlighted not only its importance to keep professionals’ skills up to date but also the need of covering more midwifery topics and move away from the concept that only training on obstetric emergencies needed regular updating:

*“(…) a number of midwife respondents felt that practicing within them required different skills and a level of confidence, which they were not well prepared for. (…) Midwifery managers and midwives in our study recommended mandatory training in normal birth skills to address this concern.” Authors,* [11]*, page 5 and 6*

*“Every year at our mandatory training, for three days (…) we have skills drills of obstetric emergencies and haemorrhage and eclamptic fits and stuck babies and breech babies and all of that, and I always, and in the feedback I always write, ‘Where’s our midwifery skills training? You assume everybody is up to speed with physiological third stage and augmenting labour naturally and advice on post-dates pregnancy etcetera … and it’s not given much value by the midwives themselves or by the people who train us or by the obstetricians.” Midwife,* [18]*, page 15*

Several studies described what they termed as *“skills hierarchy”* when planning training for maternity professionals with more attention given to the so called “*high risk skills*” and not on the skill for physiological birth. Instead, the kind of skills reported as prerequisite of working in a MU were often the ones more related to physiological birth and autonomy in decision making [11, 17–19].

### Exposure to MU model

In some studies, the importance of exposure to the MU model of care for professionals before the opening of a new MU was also discussed [17–19, 22, 23].

*“The practical part of the course was held in several institutions. (…) To begin practicing at these Birthing Centers (MUs), the required care for nurse internship at these facilities was addressed. During the internship, it was possible to learn the philosophy and administration of each of the centers. The situations experienced by the nurses reflect the different systems of care in this field that would ultimately influence the professional practice of each one of them upon returning to Brazil.” Authors,* [22]*, page 197*

The aspect of the exposure to midwifery models was not limited to other midwives but could be promoted to other maternity professionals and students too. In some contexts, where MUs were not established yet, home birth represented another option to experience midwifery led care [23]. This was important not just for witnessing the model of care but also to gain an insight in each other’s role and promote integration amongst the team.

*“Physician exposure to home birth is associated with more positive attitudes toward home births, highlighting the importance of increased exposure through interprofessional training opportunities in education and practice” Authors,* [23]*, page 547*

In countries where MUs were already established, AMU represented the middle ground to increase exposure to physiological birth to the maternity team and to consolidate autonomous midwifery care for midwives.

*“Lack of confidence in working with physiological birth was also reported by some hospital-based midwives, and the alongside midwifery unit was seen as a steppingstone to all midwives developing their skills and confidence in midwife-led care” Authors,* [18]*, page 17*

The concept of “*contamination of practice*” was also mentioned in three studies in which rotations of staff or an international exchange were applied hoping to bring back into the OU some of the MU philosophy of care [17, 18, 22].

### Promote collaborative and well integrated working relationships

In all case studies, the planning and opening of the MU involved communication, negotiation and coordination between different stakeholders within the same organisation or part of different ones. This highlights the importance of a collaborative approach to the change. When the importance of interdisciplinary work is acknowledged, included in the in-service training and constitutes part of the team vision, this aspect was found to be a significant enabler of the change [17–21, 23–25]. Conversely, the lack of an interprofessional approach could make the MU service isolated and lead to a lack of confidence and trust amongst professionals of the same team [11, 17, 18, 25].

“*Participants from all 4 hospitals described interprofessional meetings very early in the planning process, ensuring that all voices were considered in the birth center (MU) development.”* Authors, [23], page 544

Establishing a vision amongst the whole maternity team in which the MU is part of the care pathway for uncomplicated pregnancies and all professionals are on board with that seemed to be a key facilitator. Having opportunities to spend time together during training days was highlighted:

*“Participants gave several examples of interprofessional training opportunities resulting from the opening of the birth centers, including hospital drills, mock EMS (emergency medical service) dispatch calls and transports from the birth centers (MUs), welcoming students from different professions to the centers, and including center tours as part of EMS personnel orientation. These opportunities increased understanding of each other’s knowledge, training, and roles, and improved participants’ ability to communicate with one another.”* Authors, [23], page 546

This also helped the strategic planning during meetings held to gain support of the managers and organisational leadership.

In more than one occasion the need of “*compromising*” and “*negotiating*” was mentioned when discussing the change [20, 24]. This was, however, most of the times endured by the midwifery component and not by the medical staff:

“*It appeared that only the nurses gave up some of their plans. Physicians were either for or against a birthing room (MU) in general*.” Authors, [20], page 264

This illuminated an imbalanced power relationship when it comes to planning a change, even towards a model that is midwifery-led.

#### Professional relationships

The opening of a new midwifery led setting may create a separation amongst midwives and polarisation of the work. This could lead to the scenario in which midwives might be ‘othering’ colleagues for working in the other setting or for being either too medicalised or too pro-physiology. This nourished the “*them and us*” culture and constituted a main barrier to the integration of the maternity team.

*“Tensions identified among staff were mostly between midwives working in different areas, particularly alongside midwifery units and obstetric units, rather than between obstetricians and midwives.” Authors,* [18]*, page 26*

These tensions were noted and voiced not just by midwives but by managers and service users too who perceived these as potentially detrimental to the care provided [18, 20].

Rapport with obstetricians varied across the different case studies and it seemed to be related to how well midwifery led models of care were already established in the respective context. In the more recent English studies, obstetricians were overall in favour of the idea of a new MU [17], whereas in the Brazilian study a great deal of tension was reported with the medical corporation, which actively opposed the initiative of the new MU [21].

Across the studies, support from the obstetric component (whether active or passive) was found to be an important, and even fundamental, facilitator to the implementation of new MUs.

*“In fact, unless chief obstetricians positively sanctioned the idea, success would have been impossible. The involvement of the chiefs ranges from strong support for the idea to passivity that allowed nurses to make the idea reality.” Authors,* [20]*, page 263*

*“In the light of apparent tensions between midwives and doctors voiced in the NBSG (Normal Birth Strategy Group) and because communication with doctors was proving difficult a new attempt was made to gain some insight into the views and opinions of doctors. Initially doctors had not been considered primary stakeholders in midwifery-led care but as the project progressed it became clear that their cooperation in moving the project forward was fundamental.” Authors,* [24]*, page 754*

This seemed to be because midwives often need medical support to be enabled to apply changes and improvements to the service. As mentioned in theme one, gendered dynamics and the hierarchical configuration of the healthcare system play a significant part in this.

#### Integration within the service

On a similar note, when discussing the importance of a multi-layered change, the concept of integration was described as an essential feature. With the term “*integration*” studies referred to the collaboration on an organisational level between different departments of the maternity service and on a professional level between different team members.

Sometimes, the change towards a MU model of care became a useful opportunity to reflect and improve integration in the maternity services:

*“Participants described the planning, implementation, and monitoring of the birth centers as a motivating force that improved interprofessional practice between different stakeholders, including nurses, physicians, midwives, paramedics, administrators, and the regional health network.” Authors,* [23]*, page 546*

When planning the implementation of a new MU, there should be awareness that adding a new branch of the service to the current maternity layout may create, especially in the first phase, disjuncture and tensions amongst the professional team [18]. Some initiatives to overcome this barrier were mentioned: planned rotations of staff, mentoring for midwives who are less confident and promotion of case-loading models [17, 18].

Another key topic that could play the role of a barrier was the staffing level. Shortage of staff experienced was due to either a permanent lack of appropriate recruitment of midwives for the MU team, or occasional due to the “*pulling away*” of staff during shifts who were meant to work in the MU but had to cover shortage of staff in other departments like the OU [11, 17–19, 25]. The staff shortage had implications even in the service users’ perception of the service:

*“A problem highlighted during the data collection relates to a perceived shortage of staff. This has particularly serious implications for women likely to give birth at night.” Authors,* [28]*, page 525*

Factors that could help developing and planning a functional staffing model were identified in having a core team that would allow continuity of philosophy or care and consistent management of the MU even in case of emergencies and rotation of a part of the staff to allow exposure to this model of care of other midwives [11, 17, 18, 23].

“*Some initiatives for increasing integration of care were identified which could potentially mitigate the effects of creating new boundaries or discontinuities in the service. These could also support quality and safety of care, and the well-being of professionals as well as service users. They included a planned system of rotation for staff, with mentoring for midwives who are less experienced and skilled in caring for normal physiological birth and more integrated community-hospital models in which midwives based in the community attend the women on their caseload giving birth at home or in the FMU or AMU and transfer with them if required.” Authors,* [17] *, page 546*

#### Communication

Effective, respectful and appropriate communication, both verbal and non-verbal, was identified as having a central facilitator role in positive stakeholders’ relationships. In some cases, educational activities were used to solve some communication issues and this helped to pre-empt or overcome tensions amongst the team. For example:

*“We’ve identified gaps in terminology between the people talking on the phone, so we’ve been able to provide education. Yeah, it’s been very, very helpful. Had we not done that, I could see that we could have had conflicts simply because we didn’t understand each other and why we were doing things a certain way and I think we’ve been able to completely avoid that or interrupt it if it was going to start because we’ve been able to go, ‘Oh, why’d they do that?’” Paramedic,* [23]*, page 546*

The opportunity of a regular dialogue and exchange of opinions and ideas to review and debrief practice was also mentioned as important factor to improve communication between the different professional parties [17, 18, 23, 24].

Appropriate information about the MU to the service users and the definition of a clear pathway of care outlined was reported to be a key facilitator for the successful implementation:

*“Successful implementation was also dependent on a clear clinical pathway from the beginning of pregnancy until the onset of labour.” Authors,* [11]*, page 6*

Lack in providing such information and the options to the service users (both during the implementation process and later once the MU was established) was reported to have a significant impact on the implementation outcomes of accessibility and sustainability [11, 18].

However, communication with service users was not mentioned much in the studies, suggesting a lack of attention to this issue. In the Chinese and Iranian case studies, the MU was perceived as a good alternative to receive better verbal and non-verbal communication and avoid mistreatment [19, 27]. The Brazilian case study reported how an organised civilian movement for birth rights was successful in influencing the governmental spheres [21].

### Invest in different components of leadership

As shown in Table 4, those who moved forward the idea of the implementation of MU were often midwives, nurse midwives or midwifery managers highlighting the importance of the midwifery component in leadership for this type of change. Senior midwifery support was often mentioned and in the English studies this was identified in the figure of the consultant midwives [11, 17, 18, 24].

**Table 4:** Overview of different strategies used to implement the MUs

N	Country	Year	Who initiated/led the implementation	Drivers to open the MU (WHY?)	Strategy (HOW?)
1A	China (A)	2009	Researchers	Promote more humanised care to reduce intrapartum interventions and medicalisation	Engagement with leadership and training for midwives. A five-stage action research project was used to: define the plans, assess midwives’ confidence and ability, outline policies, procedures and standards of practice, review and tackle the obstacles found in the previous steps.
1B	China (B)	2009	Researchers	(See 1A)	A follow up from study 1A with the same strategies and adding the involvement of a wider range of stakeholders (including midwifery staff managers and researchers) to assess feasibility of the MU.
2	US	1991	Nurse-midwives in four different institutions	Negotiating a middle-ground service between homebirths and the medicalised OU	Eight strategies were used, described as: going it alone, compromising, getting others involved, capitalising on consumer pressure, promoting the idea of “*it’s not different*”, playing the waiting game and overcoming government regulation.
3	Iran	2013	UNFPA and the Health Centre of Sistan and Balochestan Province	Increasing accessibility to perinatal care in areas with poor access to care	Response to a local situation in which vulnerable women lacked access to appropriate care and a high birth rate to increase accessibility of facilities and reduce perinatal mortality. UNFPA supervised the first three years of operation.
4A	Brazil	2009	Brazilian Ministry of Health	Promoting more humanised care to reduce intrapartum interventions and medicalisation	Normal Childbirth Centers or Childbirth Houses were implemented as consequence of a strategic governmental initiative to reduce medicalization in childbirth in Brazil.
4B	Brazil	2013	Brazilian Ministry of Health (MoH)	(see 4A)	The MoH invested in nurse-midwives’ professional profile by sending them for an international exchange in a country where MUs were established. This was considered to give them greater symbolic power to fight for the implementation of the MU.
5	Canada	2018	The Ontario Ministry of Health and Long Term Care	Implementing evidence into practice	The availability of evidence was the reason why the MoH decided to invest in this model of care. They used interprofessional approach for planning the change, develop appropriate policies, protocols and to enhance teamwork. They also gave attention to the midwives’ admission privileges at the moment of transfer and to the continuous service evaluation.
6	England	2005	Consultant midwife	Opportunistic or pragmatic reasons such as reconfiguration of the service, including centralisation	The refurbishment of the maternity setting became the opportunity to promote the inclusion of a MU. Consultant midwife doing a postgraduate thesis initiated an action research study, which included different stakeholders (including managers midwives and medical staff) and established a group to promote normal birth.
7	England	2018	Local managers (not specified)	Implementing evidence into practice	After the publication of the Birthplace study in 2011 the NICE Intrapartum guidelines published in 2014 recommended all 4 options of birthplace. This guideline had a significant impact and was used by stakeholders as main facilitator to make the case and open new MUs nationally.
8	England	2020	Midwifery managers	Implementing evidence into practice	Key factors for successful implementation were: leadership (and continuity of it), active promotion of the MU as part of the local policy, clear clinical pathway from the beginning of pregnancy until the onset of labour and appropriate information for women.
9A 9B	England	2014 and 2018	Midwifery managers	Opportunistic or pragmatic reasons such as reconfiguration of the service, including centralisation	Key drivers for development of AMUs in all the services studied had been a combination of pragmatic, even opportunistic, decisions. Lead midwives had often seized an incidental chance to develop the service responding also to financial constraints or existing plans for service redesign or improvement, including merging of different OUs within a single service organisation.

Good leadership was sometimes shown in groups or by a single professional who could either be a senior midwife or an obstetrician depending on the context. The role of one charismatic and motivated leader was often mentioned as key ingredient to start a conversation and to initiate the adoption process.

“-*it's crucial to have an inspirational leader. If you don't have somebody at the very top who is passionate about it (MUs) happening, it won't happen. And they must cascade, get everybody onboard. – (Midwives Focus Group)*

*-a charismatic leader to kind of bring it together… unless you’ve got that then I think it’s quite hard to bring it to fruition.- (Manager)” Midwife and manager,* [11]*, page 6*

The figure of one charismatic and motivated leader was reported to be essential especially at the early stages and later, during the planning process, this leader needed to be combined with a group of stakeholders and interdisciplinary members of which the obstetric component is essential. This layer of leadership was described to be necessary for the integration of the service and for promoting a culture of inclusion of different figures (including service users) in the development of a service change:

*“Management respondents emphasised the importance of senior midwifery, obstetric and general managers working together to support and sustain the development.” Authors,* [17]*, page 24*

Overall, the studies in this review identified the key functions of leadership to support the implementation of a new MU:

- Inspire and start a conversation about the change and promote a vision
- Advocate for the team and for the service users
- Promote participation of different figures for planning and developing the change
- Ensure integration within the service
- Negotiate and move strategically with inside knowledge
- Support training and establish a learning culture

### Think physical environment

All studies discussed of concept of the MU as a distinct built environment separate from the OU as a prerequisite of an effective implementation plan. In some cases, the refurbishment of the physical environment or a reconfiguration became the means to promote a change in clinical practice and in the birth culture of the local context [17, 18, 20–22, 24, 28]. The new physical layout was the most visible feature of the wider change that was being promoted and implemented:

*“The accounts of professionals and service users suggest that these different aspects of the care environment cannot simply be unpicked as they are closely inter-related. Although some respondents regarded the design aspects of the environment, such as domestic touches, as superficial in relation to actual care processes, our study findings overall suggest that attempts to alter either processes or environment of care in isolation are less likely to be effective.”* Authors, [17], page 26

The literature reported that an appropriate use of the physical environment has the potential to be an important strategy for the new MU, especially at the beginning of the negotiations when involving different stakeholders [11, 17, 18, 24].

On the other hand, if the planning of the change does not consider all the different layers implied, including the shift in culture, practice and integration required, then there is the risk that the physical layout case alone could become a trap in which energy and resources could be wasted. Focusing just on the MU physical layout and not on the MU model of care was reported as a potential barrier to effective implementation [17, 18, 20, 24]:

*“I’m afraid we could end up with a room that’s just decorated differently; that’s about all that would be different”* Midwife, [20], page 265

The clear physical separation from the OUs was also mentioned as facilitator for the implementation of the new MU:

“*We thought it would be easier to do it outside the hospital due to institutional resistance.*” Manager, [21], page 872

And when it was not, it became an obstacle to the MU model of care:

*“As there was no physical barrier between these rooms and the rest of the labour ward, it was too easy to use them for other purposes when demand was high.” Authors,* [24]*, page 754*

## Discussion

The twelve studies included in this review were heterogeneous in their aims, methodology and local contexts but it was interesting to find agreement and coherence of many of the findings. Themes and sub-themes identified in single studies were coherent with those looking across a wider range of services [11, 17, 18, 25].

Key drivers that led to the implementation of new MUs were: desire to reduce interventions and to promote humanised care [19, 21, 22, 28], need to negotiate a middle-ground service between homebirth and OU [20], desire to increase access to care [27], commitment to implement recent scientific evidence [11, 23, 25] or opportunistic reasons such as refurbishment of the unit or reconfiguration of the service [17, 18, 24].

Few studies focused explicitly on macro-level influences such as wider culture and social influences, policies or healthcare systems structures suggesting an approach of mainly institution-centred. The systemic issues mentioned concerned the role of barriers that gendered power dynamics, hierarchy in the healthcare system and an industrialised approach in healthcare can play [11, 17, 18, 21] but only a few studies included a focus on the role of service user or public activism in implementation or examined levels of public awareness and information [17, 18, 21]. This seems to suggest that women’s groups could be big drivers in facilitating change in maternity [32, 33] but lack of their inclusion in the data collections of the selected studies shows how this aspect has not been researched enough yet on this review’s topic. We recommend that future research should involve more focus on the service users’ perspective.

In spite of differences in midwifery autonomy across the contexts of this review, most studies discussed the importance of a midwifery identity and the role that this profession has in the respective society prior to implementing a MU [11, 17–21, 23]. The ICM Standards for Midwifery Education (revised in 2021) aim to address local differences and promote a skilled professional midwifery workforce internationally to facilitate the implementation of midwifery led care models [34].

Walsh et al. (2020) noted lack of awareness of the economic evidence that MUs are cost-effective even when working at 30% of their capacity [11, 35, 36]. Different contexts showed how different commissioning systems could affect the adoption of the MU model. Most studies reported the need to adopt a cost-saving model to support a climate of financial constraint. This situation in which commissioners and managers are required to save money in the short-term was reported to be a main barrier to the implementation of MUs. Promoting the concept of cost-effectiveness among stakeholders and allowing longer-term goals to be reflected in the healthcare financing system were reported to be facilitators for this type of change [11, 17, 18].

National guidelines and local protocols were mentioned as key enablers of the change and found to play an important role in terms of “readiness” of the local context. For participants it was equally needed to have some reference at a national level (via guidelines) and on a local level (via organisational protocols). This helped the perception of safety, protection for midwives’ work, midwives’ autonomy and the sense of integration amongst professionals in the organisation. Furthermore, the quantitative results from Walsh et al. (2018) described the impact that Research and policy can have in affecting the configuration of maternity services and therefore support the implementation of MUs.

Training midwives (sometimes with the multidisciplinary team) was a common strategy to facilitate the implementation across all studies. One element reported to be relevant for promoting trust in the MU model and integration within the team was the exposure to the MU model. AMUs were seen as the appropriate middle ground to facilitate this exposure [17, 18, 25, 37]. The theme of exposure to midwifery-led care models was also mentioned in relation to midwifery students learning experience in Rawnson’s work (2010) which showed a better learning experience and the application of theory to practice when they were exposed to caseloading models [38].

All cases mentioned the importance of a collaborative approach to the change. This is coherent with work previously conducted in research about patient safety which identified lack of these components as threats to patient centred care and safety [39–41].

The professional tensions mentioned showed a clear majority of intra-professional issues more than inter-professional ones. This is coherent with feminist work on midwifery arguing that midwives could be at the same time be the “oppressed” and “oppressors” [42]. This is consistent with previous findings that identified lack of understanding and trust between midwives working in AMUs or in OUs [43, 44]. Such negative relationships have been identified as a significant cause of midwives’ stress, emotional labour and reduction in practice confidence [45–47]. Across the studies, support from the obstetric component (whether active or passive) was found to be an important facilitator to the implementation of new MUs.

This study was coherent with previous work that identified leadership as important enabler for the promotion and adoption of new MUs [9, 11, 18]. A necessary feature was the senior midwifery component, although support from and collaboration with obstetric leaders was also found to be a key enabling factor. The studies reported the relevance of both single leaders who often initiated the conversation and were key for the engagement and a group of stakeholders for moving the projects forward at later stage.

A good level of integration within the organisation was found to be a crucial facilitator. The shift from the existing maternity configuration to the inclusion of a MU could in fact either destabilise the existing structure or reinforce the rapports within the organisation [17, 18, 23].

Previous studies have shown that the physical environment in the healthcare sector, and specifically in midwifery, has the potential to affect staff wellbeing (or burnout) and therefore the care that is provided to service users [44, 47–51]. Stakeholders tend to have the greater perception of safety towards AMUs in contrast to FMUs. However, participants reported the need to be physically separated and independent to facilitate the implementation and future sustainability [17, 18, 24]. The case studies where normal birthing rooms were attempted and had closer proximity to the OU reported more effort and difficulty in doing so [20, 24]. Other authors have previously explained this concept using the theory of Birth Territory by Fahy (2008) in which AMUs were an intermediate space with more complex power dynamics and jurisdictions due to the closeness to the OU [52, 53].

### Strengths and limitations

The strengths of this review lie in the robust research approach, systematic search and critical selection of studies to meet the inclusion criteria. This review is also very specific to the phenomenon of interest of the “implementation” of new MUs, excluding confounding factors which could be related to the improvement aspect and the uptake of existing ones, although in practice this was challenging to achieve as authors often described factors as important to quality and sustainability of care after implementation. While there was considerable heterogeneity of contexts in which implementation took place, the analysis found consistencies amongst the studies. This adds value to the findings of the review, but more studies are needed in other contexts, including low-income countries. One limitation identified was that amongst the twelve studies only four had contributions from service users denoting a lack of involvement of their perspective when conducting this type of study.

### Implications for policy and practice

Our review synthesised the strategies used in different international context when attempting to implement an innovation such a midwifery unit. This synthesis helps to identify what are the drivers that usually make the MU implementation happen, the elements that could become barriers or facilitators and which strategies had been reported in the existing literature when opening new MUs. Those elements should be considered by stakeholders to optimise time and resources in future attempts to open new MUs and when preparing an implementation strategy.

This review also identifies a gap in evidence to practice around active involvement of service-users input in maternity service reorganization. Future international policies on MUs should address this gap.

## Conclusions

MUs are a valid and evidence based model of care and their implementation has been recommended by many international guidelines and studies [3, 4, 31, 54, 55]. This is the first review that examines what kind of strategies have been used when implementing new MUs in different national contexts to identify what factors should be considered when adopting such innovation. This review examines experiences of implementing MUs, analysing the strategies used so far in different national contexts. Key drivers were found to be: desire to reduce interventions and to promote humanised care, need to negotiate a middle-ground service between homebirth and OU, desire to increase access to care, commitment to implement recent scientific evidence or opportunistic reasons such as refurbishment of the unit or reconfiguration of the service. Three key themes were found to be important for the readiness of the local context and four key themes were identified in the analysis of implementation strategies.

Changing the mainstream maternity service requires time and a multi-layered change in which cultural, organisation and professional factors should be taken into consideration and addressed to promote readiness in the local context.

## Data Availability

All data and materials reported in this study are available from the corresponding author.

## Abbreviations

AMU: Alongside midwifery unit
FMU: Freestanding midwifery unit
HIC: High income countries
LMIC: Low and middle income countries
MU: Midwifery unit
MUs: Midwifery units
OU: Obstetric unit
US: United States of America
UNFPA: United Nations Population Fund
MoH: Ministry of Health
